# Transcriptome Analysis of Dimorphic Fungus *Sporothrix schenckii* Exposed to Temperature Stress

**DOI:** 10.1007/s10123-020-00136-y

**Published:** 2020-07-20

**Authors:** Dan He, Xiaowei Zhang, Song Gao, Hailong You, Yanbing Zhao, Li Wang

**Affiliations:** 1 Department of Pathogenobiology, Jilin University Mycology Research Center, College of Basic Medical Sciences, Jilin University, No. 126 Xinmin Street, Changchun, 130021 Jilin China; 2 The Laboratory of Changchun Children’s Hospital, Changchun, 130061 China; 3 Beijing ZhongKai TianCheng Bio-Technology Co. Ltd, Beijing, 101111 China; 4 Department of Pediatrics, The First Affiliated Hospital, Jilin University, Changchun, 130021 China

**Keywords:** *Sporothrix schenckii*, Dimorphic fungi, Transcriptome, Dimorphism, Virulence, Pathogenesis

## Abstract

**Purpose:**

*Sporothrix schenckii* is a thermally dimorphic fungus. In a saprotrophic environment or culturing at 25 °C, it grows as mycelia, whereas in host tissues or culturing at 37 °C, it undergoes dimorphic transition and division into pathogenic yeast cells. *S. schenckii* can cause serious disseminated sporotrichosis in immunocompromised hosts and presents an emerging global health problem. The mycelium-to-yeast transition was a consequence of the adaptive process to different environment. Some studies showed that the transition was significantly related to the virulence and pathogenesis of dimorphic fungi. However the genetic mechanisms of this complicated biological process are poorly understood.

**Method:**

Our study presented a comparative transcriptomic analysis perspective on temperature stress in a visceral isolates of *S. schenckii*, obtaining more genetic information related to dimorphic transition.

**Results:**

The 9.38 Gbp dataset was generated and assembled into 14,423 unigenes. Compared with gene and protein databases, 9561 unigenes were annotated. Comparative analysis identified 1259 genes expressed differentially in mycelium and yeast phase, and were categorized into a number of important biological processes, such as synthesis and metabolism, transmembrane transport, biocatalysis, oxidation reduction, and cellular signal transduction.

**Conclusions:**

The findings suggested that temperature-dependent transition was tightly associated with stress adaptation, growth and development, signal regulation, adhesion, and colonization, which was predicted to be related with virulence and pathogenesis. Collection of these data should offer fine-scale insights into the mechanisms of dimorphism and pathogenesis of *S. schenckii*, and meanwhile facilitate the evolutionary and function studies of other dimorphic fungi.

**Electronic supplementary material:**

The online version of this article (10.1007/s10123-020-00136-y) contains supplementary material, which is available to authorized users.

## Introduction

Thermally dimorphic fungi are a group of important evolutionarily related pathogens, including *Sporothrix schenckii*, *Histoplasma capsulatum*, *Blastomyces dermatitidis*, *Coccidioides immitis*, *Paracoccidioides brasiliensis*, *Talaromyces marneffei* (formerly known as *Penicillium marneffei*), etc. All members of this group have a special morphogenetic transition capability known as dimorphism. In saprotrophic environments or when cultured at 25 °C, dimorphic fungus grows vegetatively as mycelia, whereas in host tissues or when cultured at 37 °C, the fungus undergoes morphogenetic transition and division into pathogenic yeast (Nemecek et al. 2006; Klein and Tebbets 2007; Boyce and Andrianopoulos 2015; Gauthier 2017).

In recent years, there has been a gradual increase in the number of infections caused by dimorphic fungi leading to increased public health concern. Among such infections, sporotrichosis caused by *S. schenckii* has the highest morbidity and is capable of causing serious disseminated disease in immunocompromised hosts. *S. schenckii* is prevalent worldwide, particularly in regions of high endemicity, and can lead to epidemic outbreaks of sporotrichosis (Barros et al. 2011; López-Romero et al. 2011; Chakrabarti et al. 2015; Conceição-Silva and Morgado 2018; AuthorsSizar and Talati 2019; Queiroz-Telles et al. 2019). *S. schenckii* is therefore an emerging global health problem.

During the transition from the environment into a host, microorganisms may encounter many types of stress. To survive and maintain homeostasis, organisms must adapt and reprogram their genomic expression and regulation to respond appropriately to stressful challenges (Bruno et al. 2010; Boyce and Andrianopoulos 2015). Temperature is one of the most important signals for adaptation. Temperature-dependent morphogenetic transition appears to be crucial in the adaptation of dimorphic fungi to new host microenvironments (Rappleye and Goldman 2006; Cooney and Klein 2008; Sil and Andrianopoulos 2014). The transformation of fungal spores or mycelia into yeast cells is considered critical for virulence because in vivo yeast can adapt to evade the host immune system. Dimorphic transition has been shown to be accompanied by changes in components of the cell wall and membrane, and the differential expression of antigenic elements and virulence factors to subvert host immune defenses (Nemecek et al. 2006; Vanittanakom et al. 2006; Klein and Tebbets 2007; Boyce and Andrianopoulos 2015; Gauthier 2017). The mycelium-to-yeast transition resulted from the adaptation to environment is considered to be related to the virulence and pathogenicity of dimorphic fungi.

Currently, single-gene analysis is the main approach to study microorganism pathogenicity; however, this is not ideal for exploring the complicated mechanisms of pathogenesis. High-throughput transcriptomic sequencing analysis is a more suitable approach for investigating the complex interplay of gene expression and regulation under specific conditions or cellular phases, allowing genetic and evolutionary mechanisms to be revealed (Wang et al. 2009). Furthermore, this technique allows for transcript sequences and related information to be directly determined, which is crucial when a reference genome in unavailable.

The whole genome sequence of *S. schenckii* isolated from patient with subcutaneous sporotrichosis was reported and deposited at GenBank (Cuomo et al. 2014; Teixeira et al. 2014). However, little is still known about the potential molecular mechanisms of its dimorphic transition and pathogenicity, owing to insufficient genetic analysis and functional annotation. Recently, a few studies have reported specific genes and proteins expressed in the yeast phase of *S. schenckii* that may be associated with temperature-dependent morphogenetic transition and might affect the development of fungal infection (Aquino-Pinero and Rodriguez-del 2002; de Jesús-Berríos and Rodriguez-del 2002; Rodriguez-Caban et al. 2011; Zhang et al. 2018). Transcriptomic sequencing analysis of *S. schenckii* mycelium and yeast phase should be in favor of supplementing and consummating the genomic information, which will facilitate systematically revealing the complicated regulatory mechanisms behind dimorphism, pathogenesis, and the relationship between them.

In this study, a visceral isolates of *S. schenckii* isolated from a patient with pulmonary disseminated sporotrichosis was investigated. Based on RNA deep sequencing, gene expression in the mycelium and yeast phases was determined and compared. Transcriptomic analysis suggested that temperature-dependent morphogenetic transition of the virulent strain was tightly associated with stress adaptation, growth and development, signal regulation, adhesion, and colonization, which was predicted to be related to virulence and pathogenesis. The findings could offer insights into the mechanisms of dimorphism and pathogenesis of *S. schenckii*.

## Materials and Methods

### Fungal Strain and Culture Conditions

The fatal visceral isolates *S. schenckii* IFM41598 with thermotolerance was described previously (Tachibana et al. 1998; Tachibana et al. 2001). To obtain fresh mycelium or yeast cells, the isolates was inoculated on potato dextrose agar (PDA, Becton Dickinson, Sparks, MD, USA) or brain–heart infusion agar (BHA, Becton Dickinson), and plates were cultured at 25 ° or 37 °C for 1 week, respectively. Then mycelia were inoculated in potato dextrose broth (PDB, Becton Dickinson) and incubated aerobically at 25 °C with shaking at 110 rpm for 3 days, while yeast cells were inoculated in brain–heart infusion (BHI, Becton Dickinson), incubated aerobically at 37 °C with shaking at 180 rpm for 5 days, in order to obtain the same amount of mycelium or yeast cells. Cultures from each phase were washed with distilled water twice, and centrifuged at 12,000×*g* for 3 min. The pellets obtained were used for RNA extraction.

### RNA Extraction

Twenty milligrams of wet cells of mycelia or yeast cells were used to extract total RNA. The total RNA was extracted from each phase using Takara RNAiso Plus kit (Total RNA extraction reagent; Takara, Shiga, Japan) with three replicates for each sample following the manufacturer’s instructions. The RNA was then digested with DNase I (Takara) for 4 h. The quantity, integrity, and purity ratios of total RNA were examined with an Agilent 2100 Bioanalyzer and a Nanodrop 8000 (Thermo Scientific, Wilmington, DE, USA). Equal quantities of high-quality RNA from each dimorphic phase were used for RNA deep sequencing.

### RNA Sequencing and Read Assembly

Illumina sequencing was performed by the Biomarker Technology Company, Beijing, China. Poly(A) isolation of mRNA, paired-end library preparation, and RNA deep sequencing were conducted according to the standard Illumina methods and protocols. Two transcriptomes from the mycelium and yeast phases of *S. schenckii* were sequenced on the Illumina HiSeq™ 2000 system.

The RNA-seq reads from the mycelium and yeast libraries were assembled using the Trinity platform (http://trinityrnaseq.sourceforge.net/) as described previously. Short reads of each library were assembled into longer contigs based on their overlapping regions. Transcript sequences were produced based on the alignments of their paired-end information. Then, potential transcripts were clustered and formed the nonredundant unigene database.

### Unigene Analysis and Annotation

The open reading frames (ORFs) of unigenes were predicted by protein translation using the GetORF software (http://emboss.sourceforge.net/apps/cvs/emboss/apps/getorf.html). The longest forecasted ORF of each unigene was selected as the final ORF. Accordingly, the coding gene and the amino acid sequence of each unigene were obtained. Potential simple sequence repeat (SSR) markers were detected among the unigenes of more than 1 kb in length using the MISA software (MIcroSAtellite; http://pgrc.ipk-gatersleben.de/misa/). Single-nucleotide polymorphisms (SNPs) between the mycelium and yeast libraries were identified using the SOAPsnp software. SNPs that scored more than 30, with a depth of between × 10 and × 100, were analyzed.

The assembled unigenes were annotated based on a comprehensive set of BLAST searches designed to determine the most detailed descriptive annotation for each sequence in the mycelium or yeast phase. Sequences were also compared with sequences in diverse gene and protein databases, such as the non-redundant (Nr) protein databases and the Swiss-Prot protein database. Functional annotation of the unigenes based on the Gene Ontology (GO) database (http://www.geneontology.org) was obtained using the Blast2GO program. The WEGO software (http://wego.genomics.org.cn/cgi-bin/wego/index.pl) was then used to perform GO functional classification of all unigenes to view the distribution of gene functions among cellular components, molecular functions, and biological processes. The unigenes were also aligned to the Clusters of Orthologous Groups (COG) database (http://www.ncbi.nlm.nih.gov/COG) to predict and classify gene functions. The signal pathways were assigned to sequences from the Kyoto Encyclopedia of Genes and Genomes (KEGG) web server (http://www.genome.jp/kegg/).

### Comparison of Differentially Expressed Genes in the Dimorphic Phases

Compared with the nonredundant unigene database, using the BLAST-Like Alignment Tool (BLAT), the unigene transcription levels of the RNA-seq reads from the mycelium and yeast libraries were measured and normalized to the reads per kilobase million (RPKM) value (Mortazavi et al. 2008). The significance of differentially expressed unigenes in the mycelium and yeast phases was identified based on the IDEG6 software (http://telethon.bio.unipd.it/bioinfo/IDEG6/). The threshold of the *P* value was adjusted to account for multiple testing using the false discovery rate (FDR). Unigenes appeared to be statistically significantly differentially expressed with an FDR-adjusted *P* value < 0.01 and an absolute value of log2 (expression fold-change) ≥ 2 in RPKM between the two different libraries. The coordinate expression and the differential tendency of the unigenes were analyzed based on hierarchical cluster analysis. The differentially expressed unigenes were annotated by alignments with sequences available in the databases mentioned above.

### RNA-Seq Validation by qPCR

The expression levels of 17 differentially expressed genes were analyzed by performing quantitative real-time PCR (qPCR) with the StepOnePlus Real-Time PCR System (Applied Biosystems, USA) as a validation of RNA-seq. Specific primer pairs of each differential genes were designed using the Primer 5 software, and the specificity of them was confirmed by BLAST searches against the *S. schenckii* genome database (Table S1. Primers used in quantitative real-time PCR analysis). The amplifications were done in a 25-μL reaction with the SYBR Green PCR Master Mix (Applied Biosystems), 40-ng cDNA, and 0.75 μL of 10 μM each primer. The thermal cycling conditions were 95 °C for 15 min as an initial denaturation, followed by 40 cycles of 95 °C for 10 s and 60 °C for 30 s. The relative expression quantification of each gene was determined by the 2-ΔΔCt method. With this method, the fold changes were obtained in gene expression normalized to the 18S rRNA gene as a reliable reference control using GraphPad Prism v6.02 Software.

## Results and Discussion

### RNA Sequencing and Reads Assembly

This study presented transcriptomic sequencing analysis of the dimorphic phases of a virulent strain, *S. schenckii* IFM41598, using the Illumina platform. In total, 9.38 gigabase pairs (Gbp) of data for the mycelium and yeast libraries were generated with an average GC content of above 58% (58.97% and 58.45% for each library, respectively). After stringent quality assessment and data clearance, 3.61 and 3.58 Gbp were selected for each library. Approximately 35.6 million high-quality reads were generated (Q30 bases, those with a base quality greater than 30, accounted for 93.14% and 93.38%, respectively).

Using the assembly program, RNA-seq reads were assembled into transcripts that were subjected to cluster and assembly analysis. Finally, the nonredundant unigene database was harvested and contained a total of 14,423 unigenes with an average length of 1852.40 bp, which included 7897 unigenes (54.75%) over 1 kb in length (Table 1). The database provides more information than the predicted coding sequences available on the Broad Institute website with about 10,000 genes (http://www.broadinstitute.org/annotation/genome/sporothrix_schenckii/MultiDownloads.html).

**Table 1:** Length distribution of assembled unigenes in *S. schenckii*

Length range	Yeast unigenes	Mycelium unigenes	All unigenes
200–300	1954(15.60%)	2140(17.10%)	2195(15.22%)
300–500	2050(16.37%)	2193(17.52%)	2129(14.76%)
500–1000	2075(16.57%)	1972(15.76%)	2202(15.27%)
1000–2000	2398(19.14%)	2540(20.30%)	2755(19.10%)
2000+	4049(32.32%)	3669(29.32%)	5142(35.65%)
Total number	12,526	12,514	14,423
Total length	21,457,656	19,564,535	26,717,201
N50 length	3047	2785	3240
Mean length	1713.05	1563.41	1852.40

### Unigene Analysis and Annotation

Using the GetORF program from the EMBOSS package, analysis of genetic structure identified 14,389 (99.8%) unigenes with ORFs of an average length of 1010.67 bp, which included 5394 unigenes (37.49%) longer than 1 kb (Table S2. ORF length distribution of assembled unigenes in *S. schenckii*). Bioinformatic analysis identified 9561 unigenes (66.29%); many of which were annotated by BLAST searches against diverse gene and protein databases, including the Nr, Swiss-Prot, GO, COG, and KEGG databases (Table 2). Unigenes that could not be annotated by similarity to known genes require further analysis. The distribution of gene expression levels determined by reads per kilobase of an exon model RPKM values was used to evaluate the normality of mycelium and yeast libraries. Comparative analysis showed that there were significant differences of the differentially expressed genes in yeast and mycelium phase. Compared with the mycelium phase, 1259 unigenes presented differential expression in the yeast phase, including 830 unigenes that were upregulated, and the other 429 unigenes that were downregulated (Table S3. Top 25 upregulated transcripts associated with temperature-dependent morphogenetic transition, Table S4. Top 25 downregulated transcripts associated with temperature-dependent morphogenetic transition). This indicated that temperature-dependent morphogenetic transition could be the result of changes in the expression and regulation of numerous genes.

**Table 2:** Functional annotation of the mycelium and yeast phases of *S. schenckii*

Annotated databases	Unigenes	Mycelium vs. yeast
Swiss-Port	8244(57.16%)	872(6.04%)
Nr	9537(66.12%)	933(6.47%)
COG	4072(28.23%)	474(3.29%)
GO	5100(35.36%)	609(4.22%)
KEGG	2784(19.30%)	612(4.24%)
Total	9561(66.29%)	966(6.70%)

### Function Analysis of Differentially Expressed Genes

**Fig. 1:**
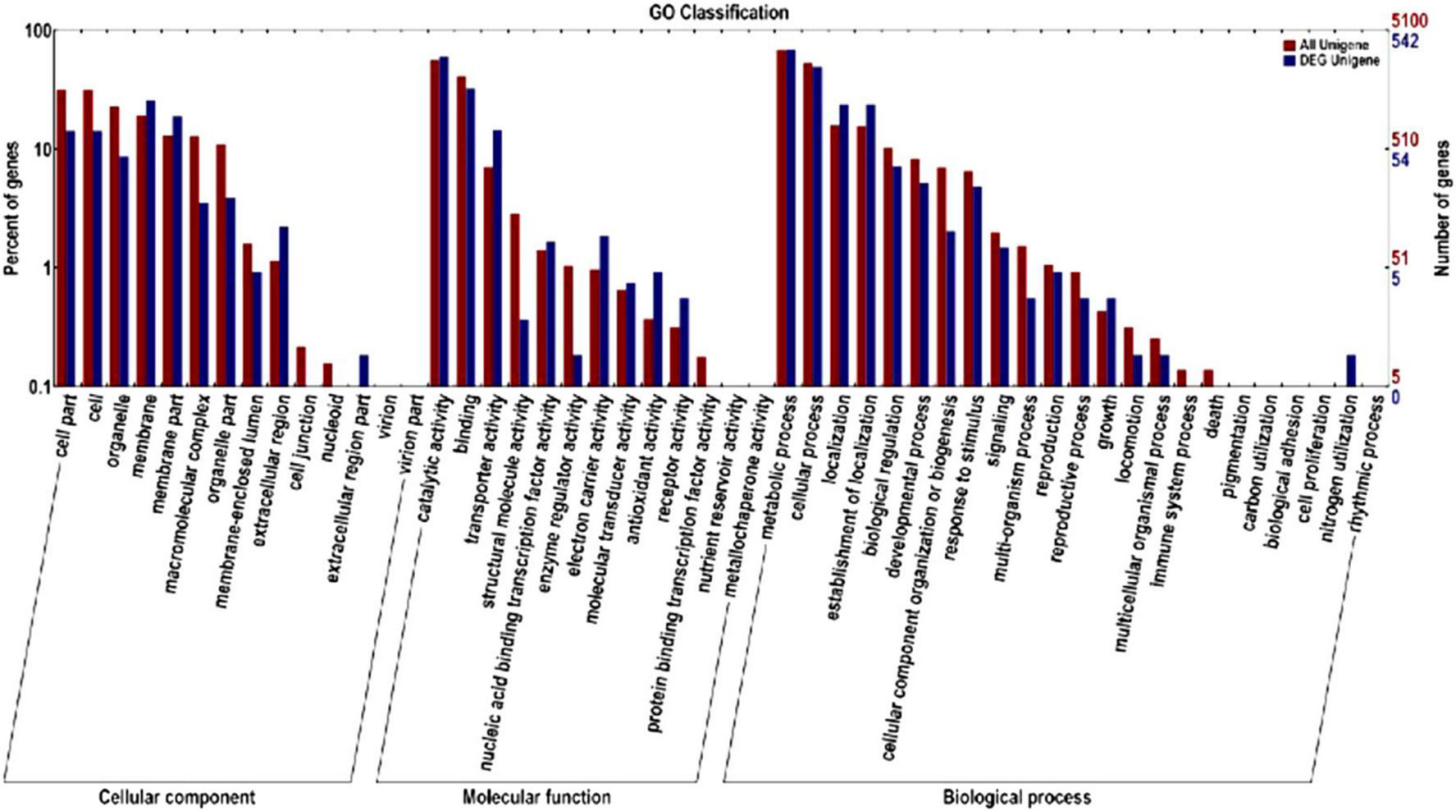
Functional annotation of the differentially expressed genes based on GO categorization

**Fig. 2:**
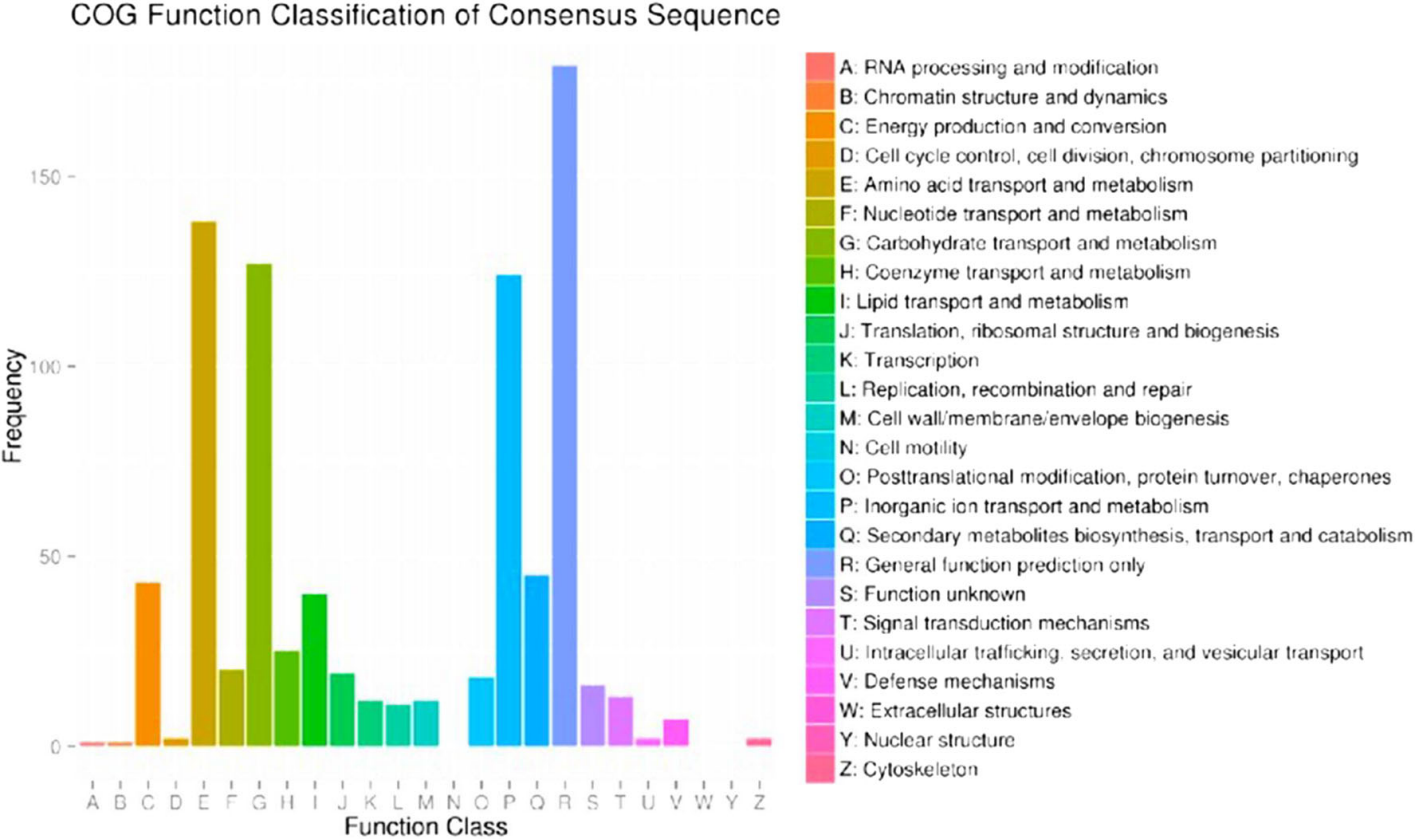
Clusters of COG classification of the differentially expressed genes in *S. schenckii*

**Fig. 3:**
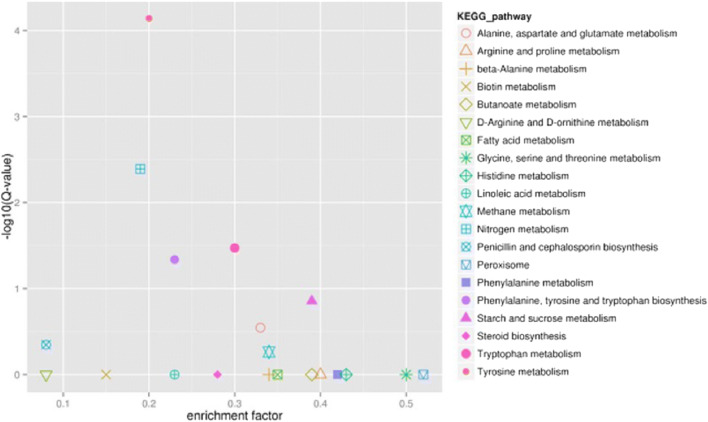
KEGG pathway enrichment scatter diagram of the differentially expressed genes in *S. schenckii*

The GO, COG, and KEGG databases were used to annotate, predict, and categorize differentially expressed genes in the dimorphic phases. GO analysis showed that 542 differential genes were significantly associated with functions such as catalytic activity, binding, and transport, involving biological processes such as metabolism, colonization, and biological regulation (Fig. 1). Functional predictions with COG database indicated that gene expression was obviously different in amino acid and inorganic ion transport and metabolism, energy production and conversion, and secondary metabolite biosynthesis (Fig. 2). KEGG pathway analysis showed that 612 differential genes were located in 79 pathways. The gene functional enrichment analysis showed that the upregulated genes were significantly enriched in metabolic pathways, such as “Starch and sucrose metabolism” (KEGG: map00500), “Tyrosine metabolism” (KEGG:map00350), and “Tryptophan metabolism” (KEGG: map00380), while the downregulated genes were enriched in “Steroid biosynthesis” (KEGG: map00100) (Fig. 3). These differentially genes might enhance the carbohydrate metabolism, signal regulation, and cell wall remodeling, which would contribute to the transition from mycelium to yeast.

These demonstrated that temperature-dependent morphogenetic transition of the virulent strain involved expression changes in a range of biological processes, such as synthesis and metabolism, transmembrane transport, biocatalysis, oxidation reduction, and cellular signal transduction. These changes were tightly associated with stress adaptation, growth and development, signal regulation, adhesion, and colonization. Since the strain used in this analysis was virulent, the changes in expression and regulation of many genes in response to temperature stress may have triggered the mycelium-to-yeast transition and the development of virulence and pathogenesis.

### Quantitative Real-Time PCR Analysis

**Fig. 4:**
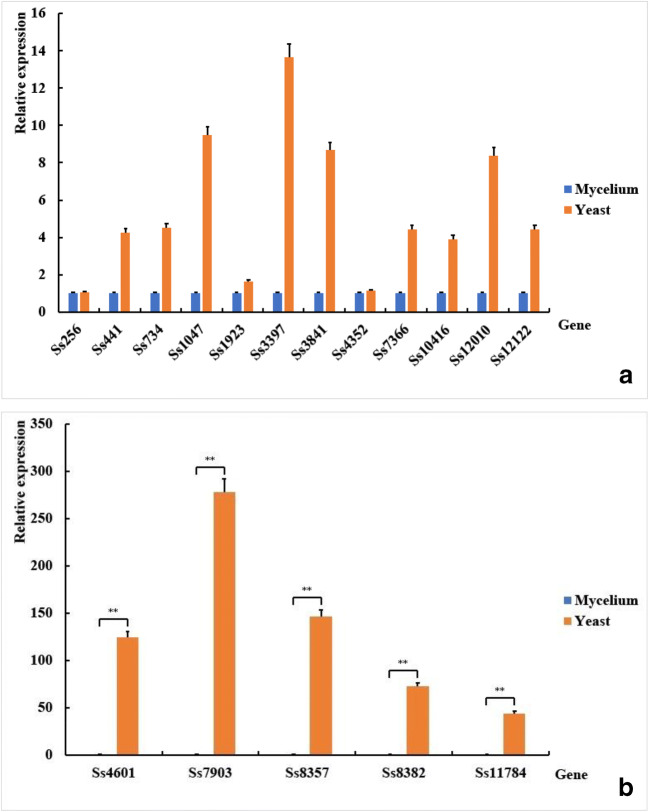
Validation of differentially expressed genes using quantitative real-time PCR (qPCR). Relative expression of 12 genes was upregulated in yeast phase of *S. schenckii* from 1 to 14 folds (a), while other 5 genes was upregulated from 44 to 278 folds (b). The data are expressed as mean ± SEM (*n* = 3), and analyzed with independent sample t tests; **P < 0.01

In order to validate the results of RNA-seq, qPCR for 17 selected differential genes was performed using independent RNA samples (Table S1. Primers used in quantitative real-time PCR analysis). The genes were choose and evaluated by qPCR considering their involvement in stress adaptation, growth and development, signal regulation, adhesion, and colonization. The correlation between the RNA-seq and qPCR results was strong that all of 17 selected genes showed the upregulation in yeast phase (Fig. 4). This analysis confirmed their transcription revealing that the differential expressed genes were really related to dimorphic transition.

### Genes Associated with Temperature-Dependent Morphogenetic Transition

#### Genes Involved in Stress Adaptation

In our study, transcriptomic analysis suggested that the expression of several heat shock proteins, such as Hsp70 family protein (SSRG_1631), Hsp90 homolog (SSRG_10557), Hsp98 (SSRG_3313), and trehalase (SSRG_3555), was found to be increased in the yeast phase, which seemed to be associated with temperature-dependent morphogenetic transition (Table 3).

**Table 3:** Partial differentially expressed genes associated with temperature-dependent morphogenetic transition

Gene ID	*S. schenckii* genome	SwissProt/NR_annotation	GO_annotation	log2FC	FDR
SSRG_256	myo-inositol-1(or 4)-monophosphatase	MFS monocarboxylate transporter [*Ophiostoma piceae*]	Inositol monophosphate phosphatase activity	3.01	1.44E-06
SSRG_441	Aspartic endopeptidase	aspartic protease [*Aspergillus saitoi*]	Hydrolase activity	3.02	1.69E-05
SSRG_477	Disintegrin and metalloproteinase domain-containing protein B	Disintegrin and metalloproteinase domain-containing protein B [*Sporothrix schenckii*]	Peptidase activity	2.27	6.88E-03
SSRG_734	Hypothetical protein	Putative uncharacterized oxidoreductase [*Schizosaccharomyces pombe*]	Oxidoreductase activity	3.23	3.16E-05
SSRG_1047	Cyclin-dependent kinase	Serine/threonine-protein kinase [*Schizosaccharomyces pombe*]	Transferase activity	3.00	1.26E-04
SSRG_1631	Hypothetical protein	Hsp70 family protein [*Ophiostoma piceae*]	–	4.49	6.81E-10
SSRG_1710	Hypothetical protein	pH-response regulator protein [*Magnaporthe oryzae*]	–	3.91	2.32E-04
SSRG_1923	Hard surface-induced protein	42 kDa endochitinase [*Trichoderma harzianum*]	Chitinase activity	3.05	1.32E-06
SSRG_3313	Non-coding region	heat shock protein hsp98 [*Neurospora crassa*]	Response to stress	2.18	9.97E-03
SSRG_3397	Catalase	catalase [*Schizosaccharomyces pombe*]	Catalase activity	4.54	2.22E-13
SSRG_3555	Alpha,alpha-trehalase	Trehalase [*Dictyostelium discoideum*]	Trehalase activity	2.77	1.46E-05
SSRG_3841	Hypothetical protein	Probable aspartic-type endopeptidase [*Aspergillus oryzae*]	Aspartic-type endopeptidase activity	4.39	1.29E-12
SSRG_4352	Spermine/spermidine synthase	Spermidine synthase family protein [*Ophiostoma piceae*]	–	5.55	0.00E+00
SSRG_4601	CBK1 kinase activator protein	CBK1 kinase activator protein [*Sporothrix schenckii*]	–	3.29	4.77E-06
SSRG_7366	Hypothetical protein	Serine/threonine-protein kinase [*Ophiostoma piceae*]	–	3.60	2.45E-06
SSRG_7903	SHO1 osmosensor	SHO1 osmosensor [*Sporothrix schenckii*]	Response to stress	2.08	8.60E-03
SSRG_8357	Salicylate hydroxylase	Aspartic-type endopeptidase [*Trichophyton verrucosum*]	Mycelium development	3.12	5.21E-07
SSRG_8371	Hypothetical protein	Cytochrome p450 oxidoreductase [*Ophiostoma piceae*]	Oxidoreductase activity	2.62	1.44E-04
SSRG_8382	Hypothetical protein	Oxidoreductase [*Ophiostoma piceae*]	Oxidoreductase activity	5.44	2.38E-13
SSRG_10074	Catalase	Catalase [*Sporothrix schenckii*]	Response to oxidative stress	1.98	6.79E-03
SSRG_10416	Hypothetical protein	Putative serine protease [*Neofusicoccum parvum*]	Serine-type peptidase activity	2.39	6.28E-04
SSRG_10557	Heat shock protein 90	Heat shock protein 90 [*Sporothrix schenckii*]	Response to stress	2.57	6.81E-05
SSRG_10583	Candidapepsin-4 precursor	Aspartic proteinase 3 [*Saccharomyces cerevisiae*]	–	3.19	8.97E-06
SSRG_11784	Histidine kinase A (phosphoacceptor) domain	Two-component system protein A [*Emericella nidulans*]	Signal transduction	3.18	1.99E-06
SSRG_12010	Hypothetical protein	Zn binuclear cluster domain containing protein [*Ophiostoma piceae*]	Chitinase activity	1.97	3.60E-03
SSRG_12122	Hypothetical protein	TOR pathway phosphatidylinositol 3-kinase [*Ophiostoma piceae*]	–	3.15	5.97E-04

Recent research indicated that the interaction of heat shock protein 90 (Hsp90) and CaMK1 could impact on the thermotolerance and dimorphic transition of *S. schenckii* (Rodriguez-Caban et al. 2011). It was reported that the expression of Hsp60 in *Histoplasma capsulatum* yeast cells increased with the adaptation to temperature stress, which was related to thermotolerance and pathogenesis (Guimaraes et al. 2011). An investigation into temperature-dependent *Candida albicans* morphogenesis indicated that elevated temperature could relieve Hsp90-mediated repression of the morphogenetic transition from yeast to filamentous growth that was crucial for virulence (Shapiro et al. 2009). A study of the thermophilic Zygomycete fungus *Rhizomucor miehei* showed that the expression of trehalase was upregulated with increasing temperature. And trehalase is considered to have an important relationship with thermotolerance and could mediate a variety of stress responses (Zhou et al. 2014). Therefore, the upregulation of the thermal stress-related proteins found in our study maybe associated with the virulence of *S. schenckii*.

With our transcriptomic data, the expression of other stress-related proteins, such as catalase (SSRG_3397, SSRG_10074), oxidoreductase (SSRG_734, SSRG_8371, SSRG_8382), high osmolarity signaling protein (SSRG_7903), and pH-response regulator protein (SSRG_1710), was also upregulated in the yeast phase (Table 3).

In many yeasts and filamentous fungi, catalases play an important role in the defense against oxidative stress and reactive oxygen species, and they have been associated with removing oxygen radicals, regulating the cell cycle, and reducing cell aging (Morano et al. 2012). The catalase of *Aspergillus fumigatus* was reported to resist the lethal effects of host phagocytes, which in part was responsible for the virulence of this organism (Shibuya et al. 2006). Oxidoreductases were found to be upregulated in yeast, and they may be important in the defense against oxidative stress in *Coccidioides immitis* according to transcriptomic analysis (Viriyakosol et al. 2013). Furthermore, researchers found that the histidine kinases TrkA and SlnA of *Talaromyces marneffei* had the ability to resist H_2_O_2_ challenge and were also associated with adaptive responses to osmotic pressure changes (Boyce et al. 2011). These findings suggested that the above genes in our study would potentially protect cells against different environmental challenges and play a crucial role in dimorphic transition.

#### Genes Involved in Growth and Development

It is generally considered that the cell wall is likely to be the first cellular structure that comes into intimate contact with the host (Langner and Gohre 2016). Chitin is an essential component of the cell wall of filamentous fungi. Chitinase and its interaction play an important physiological role in the growth. In our study, the expression of chitinase (SSRG_1923, SSRG_12010), spermidine synthase (SSRG_4352), and cyclin-dependent kinase (SSRG_1047) in the yeast phase was upregulated, which may affect cell growth and cell cycle regulation (Table 3).

Analysis of *Aspergillus nidulans* showed that the chitinase gene *chiA* could regulate cell wall remodeling and the cell cycle (Yamazaki et al. 2008). Spermidine synthase was associated with the development of mycelia and cell cycle regulation in some filamentous fungi. Studies of *Talaromyces marneffei* showed that differential expression of the S-adenosylmethionine decarboxylase gene *sadA* and the histone acetyltransferase enzyme gene *rttA* in the spermidine synthesis pathway during the dimorphic phases led to cell structure changes and mycelium-to-yeast transition, which was confirmed to effect pathogenicity (Pongpom et al. 2005; Kummasook et al. 2013). The thermostable chitinase II of a thermophilic fungus was considered to contribute to stability at temperature, over a wide range of pH (Khan et al. 2015). So the changes in these differentially expressed genes in our study might be related to dimorphic transition, resulting in increased virulence.

#### Genes Involved in Signal Regulation

Protein kinases are considered to play a major role in the regulation of cell signaling in pathogenic fungi. Our study showed that the expression of many protein kinases, such as serine/threonine protein kinase (SSRG_7366), histidine kinase (SSRG_11784), phosphatidylinositol 3-kinase (SSRG_12122), inositol monophosphatase (SSRG_256), and CBK1 kinase activator protein (SSRG_4601) was upregulated (Table 3).

The hybrid histidine kinase SLN1 of *Saccharomyces cerevisiae* is important in two-component signaling systems, which are related to environmental stress adaption, growth and development, cell wall remodeling, virulence factor expression, and drug sensitivity (Bahn 2008; Fassler and West 2010). Gene knockout or RNA silencing of the hybrid histidine kinase DRK1 in *Blastomyces dermatitidis* or *Histoplasma capsulatum* resulted in stagnation at the vegetative mycelium phase, impaired synthesis of cell wall alpha-1,3-glucan, and deficiency of the yeast phase-specific factor *Blastomyces* adhesin BAD1, calcium-binding protein CBP1, α-glucan synthase AGS1, and cell wall protein YPS3, which effected the dimorphism and virulence of dimorphic fungi (Batanghari et al. 1998; Bohse and Woods 2005; Nemecek et al. 2006; Klein and Tebbets 2007; Holbrook and Rappleye 2008; Boyce and Andrianopoulos 2015).

In the yeast phase of *S. schenckii*, over expression of DRK1 was considered to be involved in regulation of the mycelium-to-yeast transition and required for pathogenesis (Zhang et al. 2018). In addition, the calcium/calmodulin kinase could regulate transcription factors activating downstream cAMP and mitogen-activated protein kinase (MAPK) signal transduction pathways involved in dimorphic transition and yeast cell cycle regulation (Valle-Aviles et al. 2007; Rodriguez-Caban et al. 2011; Boyce and Andrianopoulos 2015). Consequently, the upregulated proteins in our study may effect on the regulation of cell signaling, and potentially on dimorphism and pathogenesis of *S. schenckii*.

#### Genes Involved in Adhesion and Colonization

Many studies suggested that enzymes related to adhesion and colonization also play an important role in pathogenesis. In this study, the overexpression of serine proteases (SSRG_10416), aspartic proteases (SSRG_441, SSRG_10583), aspartic-type endopeptidase (SSRG_3841, SSRG_8357), and metalloproteinases (SSRG_477) would be beneficial to enhance adhesion and host colonization (Table 3).

In *Histoplasma capsulatum* yeast cells, secreted CBP1 shows resistance to the phagolysosomes of macrophages and enhances colonization in the lungs, and YPS3 promotes dissemination from the lungs to systemic tissues (Batanghari et al. 1998; Bohse and Woods 2005; Klein and Tebbets 2007; Holbrook and Rappleye 2008). In the yeast phase in *Blastomyces dermatitidis* and *Histoplasma capsulatum*, beta-1,3-glucan in the cell wall was associated with immunologic recognition (Nemecek et al. 2006; Klein and Tebbets 2007). But alpha-1,3-glucan increased with the overexpression of AGS1, and beta-1,3-glucan reduced. These outcomes were favorable for adhesion and colonization in the lungs, as determined by a mouse infection model (Klein 2000; Holbrook and Rappleye 2008). Therefore, the overexpression of above proteins would promote the invasion of host systemic tissues, and was predicted to enhance the pathogenicity of this virulent strain of *S. schenckii*.

In addition, the analysis showed found the coding genes of some proteins such as ATPase family protein, glucanase, glucosidase, lactose regulatory protein, and glucose transporter were upregulated in the yeast form. And others such as sterol regulatory protein, transcriptional activator protein, and hypothetical protein in mycelium development were downregulated in the yeast form. These differentially genes may contribute to the transition from mycelium to yeast. The possible regulatory mechanisms need to be further analyzed in the subsequent studies.

Transcriptomic analysis also indicated that some homologous genes existed in other dimorphic fungi similar to the differentially expressed genes of *S. schenckii*, such as the catalase gene of *Paracoccidioides brasiliensis*, the chitinase gene of *Coccidioides posadasii*, and the spermidine synthase gene of *Paracoccidioides brasiliensis* and *Talaromyces marneffei*. However, the function of these genes has not been fully defined and their potential role in dimorphism and pathogenesis remains to be determined. Function analysis of the differentially related genes in *S. schenckii* could facilitate similar studies of other dimorphic fungal genes and genomes.

## Conclusions

This study is to reveal the transcriptomic changes of dimorphic fungus *S. schenckii* in response to shifts in temperature. Our data could offer significant insights into several interesting aspects of how fungal cells perceive and respond to stress. The findings suggest that dimorphism of *S. schenckii* is a complex biological process, regulated by many temperature-dependent related genes and signal pathways. The process also appears to be tightly associated with stress adaptation, growth and development, signal regulation, adhesion, and colonization, which is predicted to be related to the development of virulence and the process of pathogenesis. As well as providing insight into the mechanisms of dimorphism and pathogenesis of *S. schenckii*, these findings should be beneficial for similar genetic or genomic studies of other dimorphic fungi.

## Electronic supplementary material

ESM 1 (DOC 149 kb)
