# Supplementary material for: Transcriptome Analysis of Dimorphic Fungus Sporothrix schenckii Exposed to Temperature Stress
Source: Int Microbiol. 2020 Jul 20;24(1):25–35. doi: 10.1007/s10123-020-00136-y (PMC7873001; doi:10.1007/s10123-020-00136-y)
Supplement: Supplementary file 1 — (DOC 149 kb) [file 10123_2020_136_MOESM1_ESM.doc]

Table S1. Primers used in quantitative real-time PCR analysis.

| Gene ID | Protein description | Primer Sequence (5'-3') | |
| --- | --- | --- | --- |
|  | 18S ribosomal RNA | 18S-F | CGGCCCTTAAATAGCCCGGTC |
|  |  | 18S-R | ACTCCCCTGAGCCAGTCCG |
| SSRG_256 | myo-inositol-1(or 4)-monophosphatase | Ss256f | CAGGAAAAGGACAGCTCGGT |
|  |  | Ss256r | CTCTTCGCCGAGAAACTGGT |
| SSRG_441 | aspartic endopeptidase | Ss441f | TCCCAAGCAGGCTATTGAGC |
|  |  | Ss441r | TCTCGACCGGTGTCTTTTGG |
| SSRG_734 | hypothetical protein | Ss734f | GACGGTCAAGAGAGTGGTCG |
|  |  | Ss734r | TCAGCGCCCGTGTATGTAAA |
| SSRG_1047 | cyclin-dependent kinase | Ss1047f | CACAGTATTGAGCGCGAAGC |
|  |  | Ss1047r | TACCGCAAGTAGGGCATCAC |
| SSRG_1923 | hard surface-induced protein | Ss1923f | GGGTACACTCCCAGATGCAG |
|  |  | Ss1923r | ATCTCAATCGCCACGTCCTG |
| SSRG_3397 | catalase | Ss3397f | ACCGCAACGTGCAAAACTAC |
|  |  | Ss3397r | GTCCGTGTACGAGAAGAGGC |
| SSRG_3841 | hypothetical protein | Ss3841f | CACATACACGGCCAACTCCT |
|  |  | Ss3841r | TGCATTTTCGAGAGGGCTGT |
| SSRG_4352 | spermine/spermidine synthase | Ss4352f | TCGGGCGTGGATGATTTGAT |
|  |  | Ss4352r | TTCGGCAAAGCGGAAAAAGG |
| SSRG_4601 | CBK1 kinase activator protein | Ss4601f | CTCTTCCCCTGTGGGTTCAC |
|  |  | Ss4601r | GGACCTTGGTGAGCAATCCA |
| SSRG_7366 | hypothetical protein | Ss7366f | CCGTCAAGGACATGGTCCAA |
|  |  | Ss7366r | ACTTCGTTGGAGAAGCCCAG |
| SSRG_7903 | SHO1 osmosensor | Ss7903f | AGAACATGGCTGGTGCCTAC |
|  |  | Ss7903r | AGGCGATGAGTTCTCGAAGC |
| SSRG_8357 | salicylate hydroxylase | Ss8357f | ACTACAACACGCCGTACCAG |
|  |  | Ss8357r | TACGGTTGCTGGCCATAGAG |
| SSRG_8382 | hypothetical protein | Ss8382f | GCGCAGGACATTAAAGAGCG |
|  |  | Ss8382r | CACGCCTCGAAATCGGGATA |
| SSRG_10416 | hypothetical protein | Ss10416f | CAGCTTCCTCTTCCCACCTG |
|  |  | Ss10416r | TAGCGTCTGTGTCGTCAACC |
| SSRG_11784 | histidine kinase A | Ss11784f | AAACGAAGAACTGCGTTGGC |
|  |  | Ss11784r | TGTACTGCACGCAGGTTGAT |
| SSRG_12010 | hypothetical protein | Ss12010f | GCCAGGTCAAGTCCATCCTC |
|  |  | Ss12010r | AGAAGACGAGGAAAGCAGCC |
| SSRG_12122 | hypothetical protein | Ss12122f | AGAAGCAAAGCCGTCCTACC |
|  |  | Ss12122r | TTGTGTTGGGGTGTCGTCAA |

Table S2. ORF length distribution of assembled unigenes in *S. schenckii*.

| Length range | Unigene ORF |
| --- | --- |
| 0-300 | 4,843(33.66%) |
| 300-500 | 1,864(12.95%) |
| 500-1000 | 2,288(15.90%) |
| 1000-2000 | 3,273(22.75%) |
| 2000+ | 2,121(14.74%) |
| Total number | 14,389 |
| Total length | 14,542,596 |
| N50 length | 1,833 |
| Mean length | 1010.67 |

.

|  |  |
| --- | --- |
|  |  |
|  |  |
|  |  |
|  |  |
|  |  |
|  |  |
|  |  |
|  |  |
|  |  |

*Undetermined SSR due to compounding

Table S3. Top 25 up-regulated transcripts associated with temperature-dependent morphogenetic transition.

| **Rank** | **Gene ID** | ***S. schenckii* genome** | **SwissProt/**  **NR_annotation** | **GO_annotation** | **log2FC** | **FDR** |
| --- | --- | --- | --- | --- | --- | --- |
| 1 | SSRG_3349 | hypothetical protein | Uncharacterized transporter [*Schizosaccharomyces pombe*] | integral component of membrane | 8.23 | 0 |
| 2 | SSRG_3323 | hypothetical protein | hypothetical protein [*Ophiostoma piceae*] | -- | 8.11 | 0 |
| 3 | SSRG_3363 | hypothetical protein | hypothetical protein [*Grosmannia clavigera*] | -- | 8.09 | 0 |
| 4 | SSRG_3754 | triacylglycerol lipase | Lipase 5 [*Candida rugosa*] | sterol esterase activity | 7.48 | 0 |
| 5 | SSRG_1067 | sirQ protein | sirq protein [*Ophiostoma piceae*] | -- | 7.30 | 1.55E-15 |
| 6 | SSRG_3667 | hypothetical protein | hypothetical protein [*Ophiostoma piceae*] | -- | 7.09 | 0 |
| 7 | SSRG_4535 | hypothetical protein | Translation initiation factor IF-2 [*Rhodococcus opacus*] | primary metabolic process | 6.98 | 0 |
| 8 | SSRG_2803 | hypothetical protein | cell surface flocculin [*Saccharomyces cerevisiae*] | -- | 6.75 | 1.44E-15 |
| 9 | SSRG_11233 | hypothetical protein | hypothetical protein [*Geomyces destructans*] | -- | 6.63 | 0 |
| 10 | SSRG_1792 | siderophore iron transporter | Flocculation protein [*Saccharomyces cerevisiae*] | transporter activity | 6.62 | 1.11E-16 |
| 11 | SSRG_3504 | hypothetical protein | Probable 4-hydroxy-2-oxoglutarate aldolase [*Emericella nidulans*] | metabolic process | 6.40 | 0 |
| 12 | SSRG_9613 | hypothetical protein | hypothetical protein [*Grosmannia clavigera*] | -- | 6.36 | 2.22E-16 |
| 13 | SSRG_761 | sugar transporter | Maltose permease [*Saccharomyces cerevisiae*] | integral component of membrane | 6.27 | 5.57E-11 |
| 14 | SSRG_11013 | hypothetical protein | Muc1p [*Saccharomyces cerevisiae*] | -- | 6.25 | 2.42E-13 |
| 15 | SSRG_4429 | transaldolase | transaldolase [*Ophiostoma piceae*] | -- | 6.22 | 0 |
| 16 | SSRG_1371 | hypothetical protein | hypothetical protein [*Fusarium oxysporum f. sp. cubense*] | -- | 6.15 | 2.33E-15 |
| 17 | SSRG_3152 | hypothetical protein | dna repair and transcription factor [*Ophiostoma piceae*] | binding | 6.12 | 0 |
| 18 | SSRG_4486 | hypothetical protein | Dextranase [*Penicillium minioluteum*] | extracellular region, dextranase activity | 6.00 | 2.22E-16 |
| 19 | SSRG_1331 | hypothetical protein | unnamed protein product [*Aspergillus niger*] | -- | 5.99 | 1.29E-13 |
| 20 | SSRG_3997 | hypothetical protein | hypothetical protein [*Pseudocercospora fijiensis*] | -- | 5.97 | 0 |
| 21 | SSRG_11745 | amino acid permease | Uncharacterized amino-acid permease [*Schizosaccharomyces pombe*] | amino acid transmembrane transport | 5.94 | 1.11E-15 |
| 22 | SSRG_1068 | solute carrier family 20 (sodium-dependent phosphate transporter) | Phosphate-repressible phosphate permease [*Neurospora crassa*] | -- | 5.94 | 0 |
| 23 | SSRG_3451 | hypothetical protein | predicted protein [*Trichoderma reesei*] | -- | 5.92 | 0 |
| 24 | SSRG_1521 | siderophore iron transporter | trichothecene efflux pump [*Ophiostoma piceae*] | -- | 5.89 | 2.08E-09 |
| 25 | SSRG_2247 | hypothetical protein | zinc finger C2H2-like protein [*Myceliophthora thermophila*] | -- | 5.87 | 4.6E-14 |

Table S4. Top 25 down-regulated transcripts associated with temperature-dependent morphogenetic transition.

| **Rank** | **Gene ID** | ***S. schenckii* genome** | **SwissProt/**  **NR_annotation** | **GO_annotation** | **log2FC** | **FDR** |
| --- | --- | --- | --- | --- | --- | --- |
| 1 | SSRG_11354 | hypothetical protein | Uncharacterized protein [*Schizosaccharomyces pombe*] | transmembrane transport | -8.94 | 0 |
| 2 | SSRG_11322 | hypothetical protein | oligopeptide transporter [*Ophiostoma piceae*] | transmembrane transport | -8.19 | 0 |
| 3 | SSRG_4799 | hypothetical protein | C6 zinc finger domain-containing protein [*Colletotrichum gloeosporioides*] | transcription | -8.18 | 0 |
| 4 | SSRG_11285 | protein kinase domain containing protein | protein kinase domain-containing protein [*Ophiostoma piceae*] | transferase activity | -8.02 | 0 |
| 5 | SSRG_10874 | hypothetical protein | hypothetical protein [*Gaeumannomyces graminis var. tritici*] | -- | -7.83 | 0 |
| 6 | SSRG_11696 | allophanate hydrolase | glutamyl-trna amidotransferase subunit a [*Ophiostoma piceae*] | catalytic activity | -6.96 | 0 |
| 7 | SSRG_12107 | NCS1 allantoate transporter | Uncharacterized permease [*Schizosaccharomyces pombe*] | nucleobase transmembrane transporter activity | -6.54 | 0 |
| 8 | SSRG_3400 | hypothetical protein | hypothetical protein [*Verticillium dahliae*] | -- | -6.50 | 1.11E-16 |
| 9 | SSRG_5721 | hypothetical protein | Transcriptional activator protein [*Saccharomyces cerevisiae*] | transcription | -6.50 | 0 |
| 10 | SSRG_11022 | MFS transporter | Inorganic phosphate transporter [*Saccharomyces cerevisiae*] | inorganic phosphate transmembrane transporter activity | -6.46 | 0 |
| 11 | SSRG_12255 | nuclease S1 | Nuclease PA3 [*Penicillium sp.*] | nucleic acid binding | -6.42 | 0 |
| 12 | SSRG_6573 | GABA-specific permease | GABA-specific permease [*Saccharomyces cerevisiae*[ | amino acid transmembrane transport | -6.33 | 0 |
| 13 | SSRG_7862 | hypothetical protein | Glucose transport transcription regulator RGT1 [*Vanderwaltozyma polyspora*] | transcription | -6.18 | 0 |
| 14 | SSRG_11123 | hypothetical protein | taurine catabolism dioxygenase [*Ophiostoma piceae*] | oxidoreductase activity | -6.12 | 0 |
| 15 | SSRG_10906 | Cupin domain protein | cupin domain protein [*Ophiostoma piceae*] | -- | -6.09 | 0 |
| 16 | SSRG_7125 | hypothetical protein | hypothetical protein F503_03208 [*Ophiostoma piceae*] | -- | -5.98 | 0 |
| 17 | SSRG_10369 | MFS aflatoxin efflux pump | Putative HC-toxin efflux carrier TOXA [*Cochliobolus carbonum*] | integral component of membrane | -5.91 | 0 |
| 18 | SSRG_403 | nucleobase:cation symporter-1 | Allantoin permease [*Saccharomyces cerevisiae*] | -- | -5.82 | 1.39E-14 |
| 19 | SSRG_4339 | KUP system potassium uptake protein | potassium transporter [*Ophiostoma piceae*] | -- | -5.74 | 5.55E-16 |
| 20 | SSRG_7991 | hypothetical protein | hypothetical protein FOXB_07681 [*Fusarium oxysporum*] | ammonium transmembrane transporter activity | -5.74 | 4.15E-14 |
| 21 | SSRG_7268 | hypothetical protein | hypothetical protein [*Grosmannia clavigera*] | -- | -5.67 | 0 |
| 22 | SSRG_10742 | nitrite reductase (NAD(P)H) large subunit | Nitrite reductase [NAD(P)H] [*Emericella nidulans*] | nitrite reductase [NAD(P)H] activity | -5.66 | 1.33E-15 |
| 23 | SSRG_7176 | hypothetical protein | Epa5p [*Candida glabrata*] | -- | -5.62 | 0 |
| 24 | SSRG_12117 | dihydropyrimidinase | Dihydropyrimidinase [*Lachancea kluyveri*] | hydrolase activity | -5.56 | 0 |
| 25 | SSRG_4352 | spermine/spermidine synthase | spermine spermidine synthase family protein [*Ophiostoma piceae*] | -- | -5.55 | 0 |
